# Supplementary material for: Effect of climate on traits of dominant and rare tree species in the world’s forests
Source: Nat Commun. 2025 May 22;16:4773. doi: 10.1038/s41467-025-59754-7 (PMC12098762; doi:10.1038/s41467-025-59754-7)
Supplement: Supplementary file 1 — Supplementary Information [file 41467_2025_59754_MOESM1_ESM.pdf]

Supplementary Information to manuscript "Effect of climate on traits of dominant and rare tree species in the world's forests"

Figures

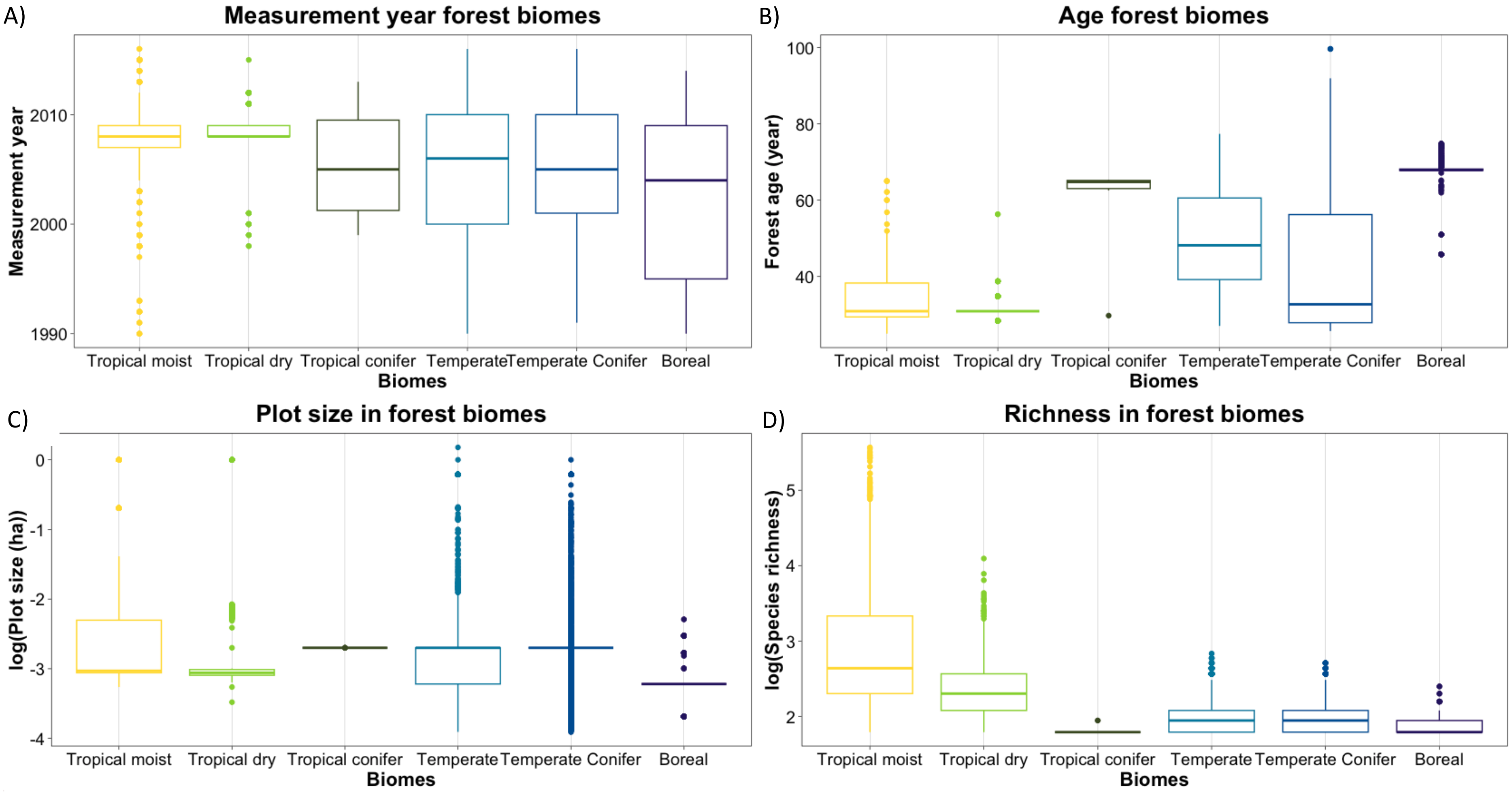

Figure S1. The range in measurement year (A), forest age (B), plot size (C) and species richness (D) in the six different forest biomes: tropical moist forest (N=1694), tropical dry forest (N=1015), tropical conifer forest (N=20), temperate forest (N=9721), temperate conifer forest (8813) and boreal forest (1562). The median, 25th and 75th percentiles of the data are visualised with the boxplot, while the whiskers present the main data range.

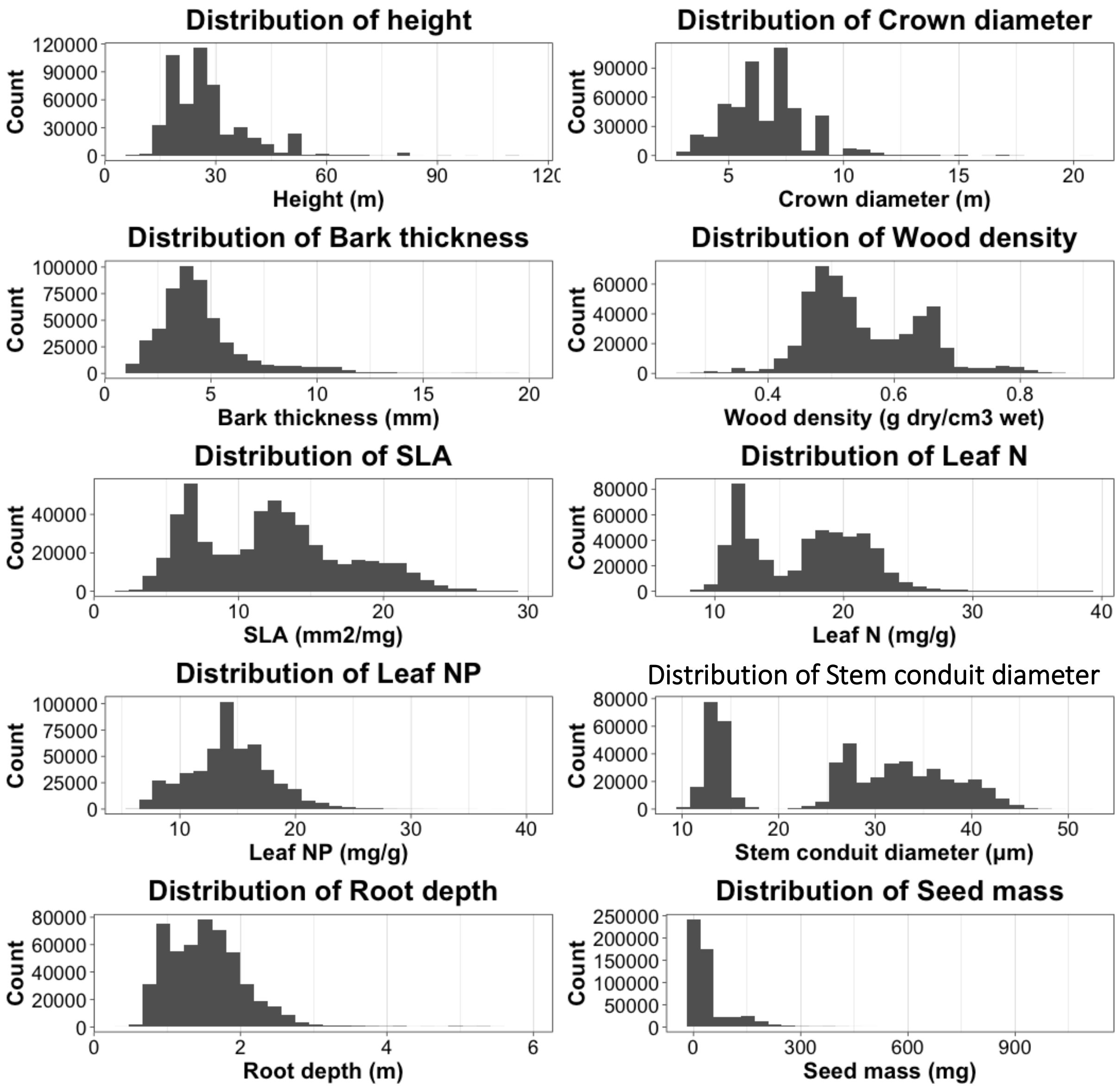

Figure S2. The distribution of the 10 imputed traits considered in this study. At the y-axis the frequency of the trait values is displayed.

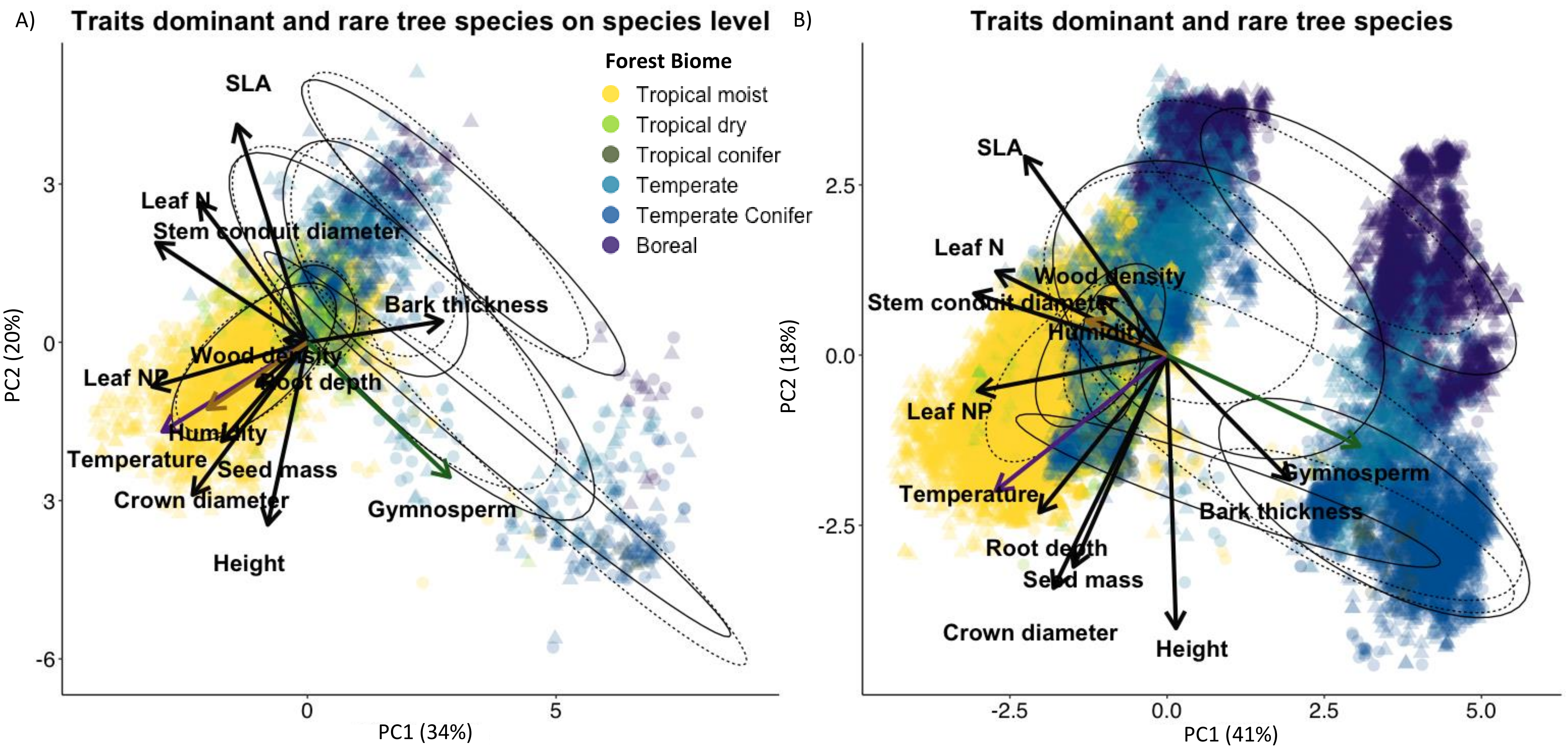

Figure S3. A PCA visualizing 10 traits of dominant species (circles) and rare species (triangles). The climatic variables temperature (purple arrow) and water availability (brown arrow), and gymnosperms (green arow) are indicated with an arrow. The six different forest biomes are visualized with different colours, see the legend for the colour explanation. The ellipses indicate 68% of the dominant and rare trait values in the different biomes. The dominant trait values

are indicated with a solid circle, while the rare trait values are indicated with a dashed line. The cluster on the left is dominated by angiosperms, while the cluster on the right is dominated by gymnosperms. A) The mean trait values of the dominant and rare species are visualised in this graph, and the circles and triangles represent therefore individual species. For the same figure on individual tree level, see Fig. 2. B) The dominant and rare species are here defined as the 10% most dominant and rare species based on the number of stems in the plot.

A)

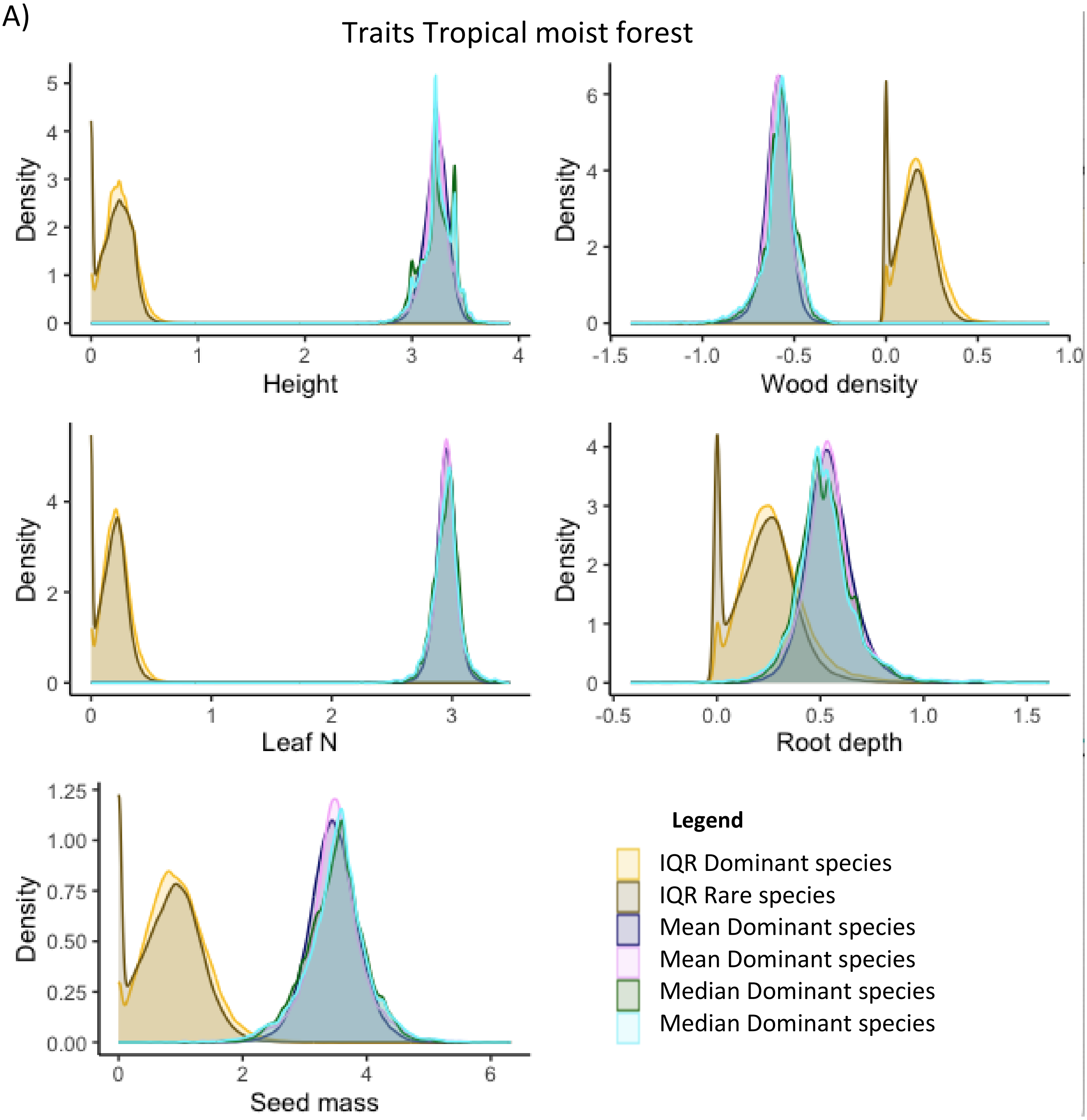

Traits Tropical moist forest
Density
Height
Wood density
Leaf N
Root depth
Seed mass
Legend
IQR Dominant species
IQR Rare species
Mean Dominant species
Mean Dominant species
Median Dominant species
Median Dominant species

B)

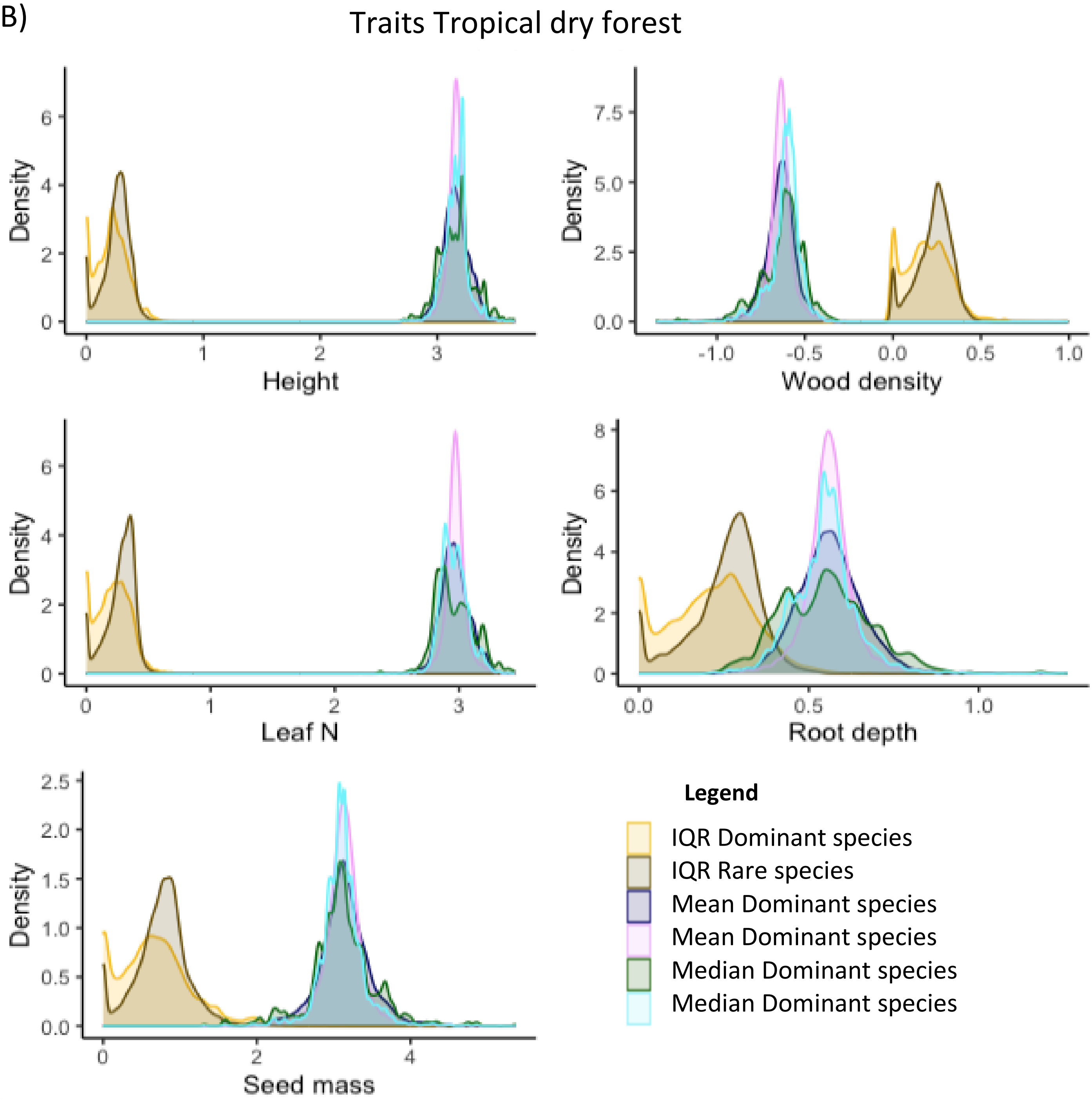

Traits Tropical dry forest
Density
Height
Wood density
Leaf N
Root depth
Seed mass
Legend
IQR Dominant species
IQR Rare species
Mean Dominant species
Mean Dominant species
Median Dominant species
Median Dominant species

C)

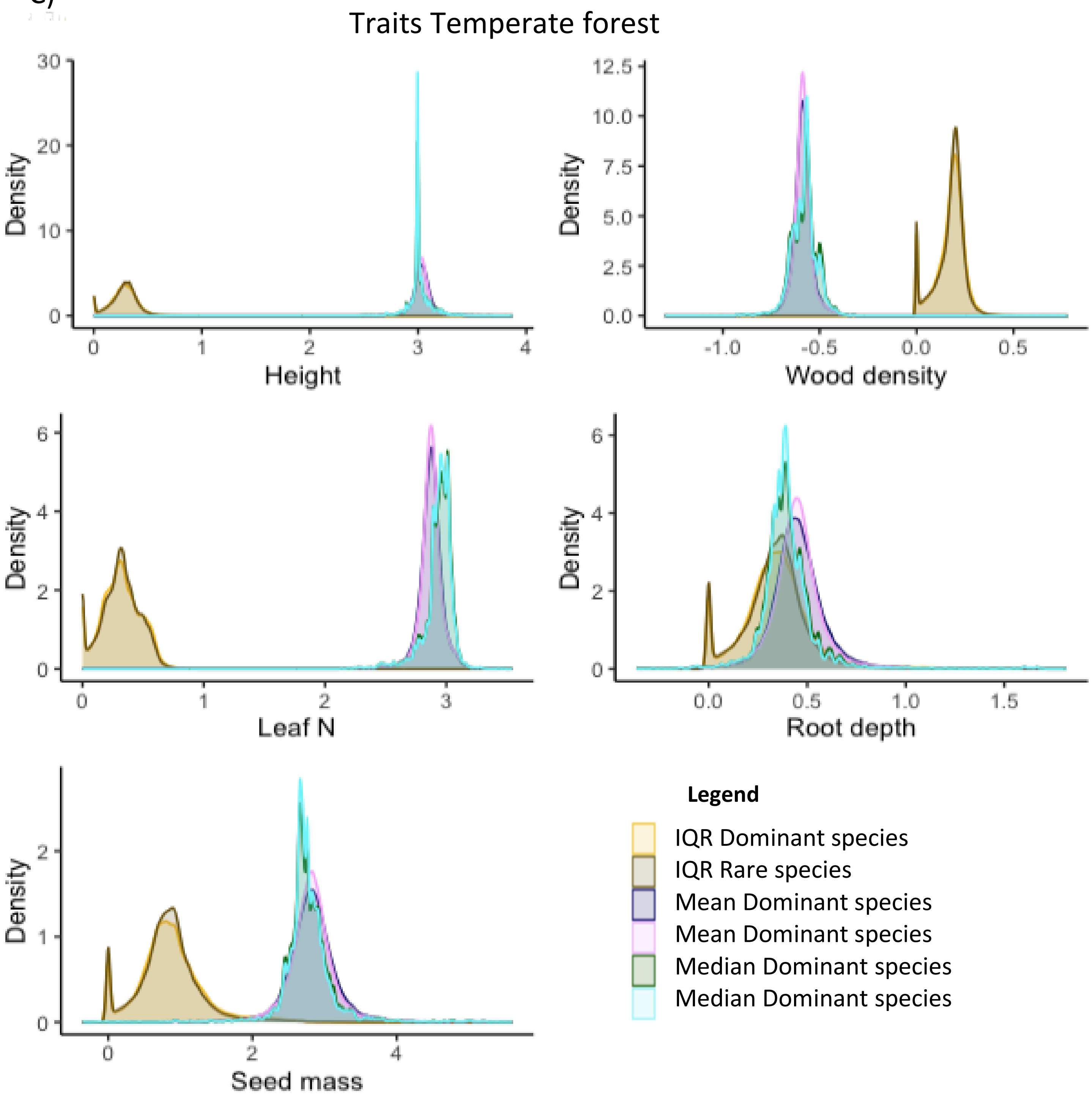
Traits Temperate forest
Density
Height
Density
Wood density
Density
Leaf N
Density
Root depth
Density
Seed mass
Legend
IQR Dominant species
IQR Rare species
Mean Dominant species
Mean Dominant species
Median Dominant species
Median Dominant species

D) Traits Temperate conifer forest

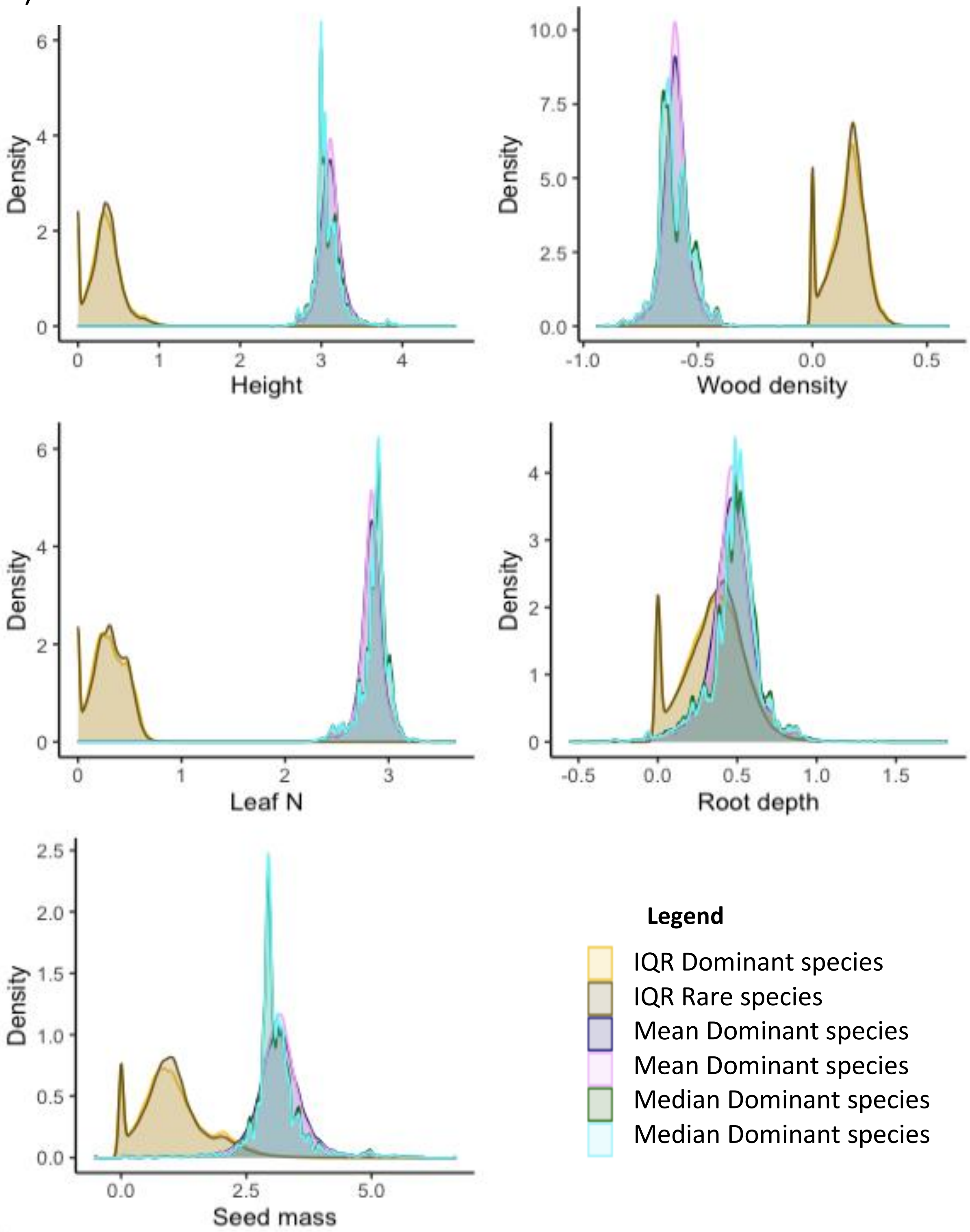

E)

Traits Boreal forest

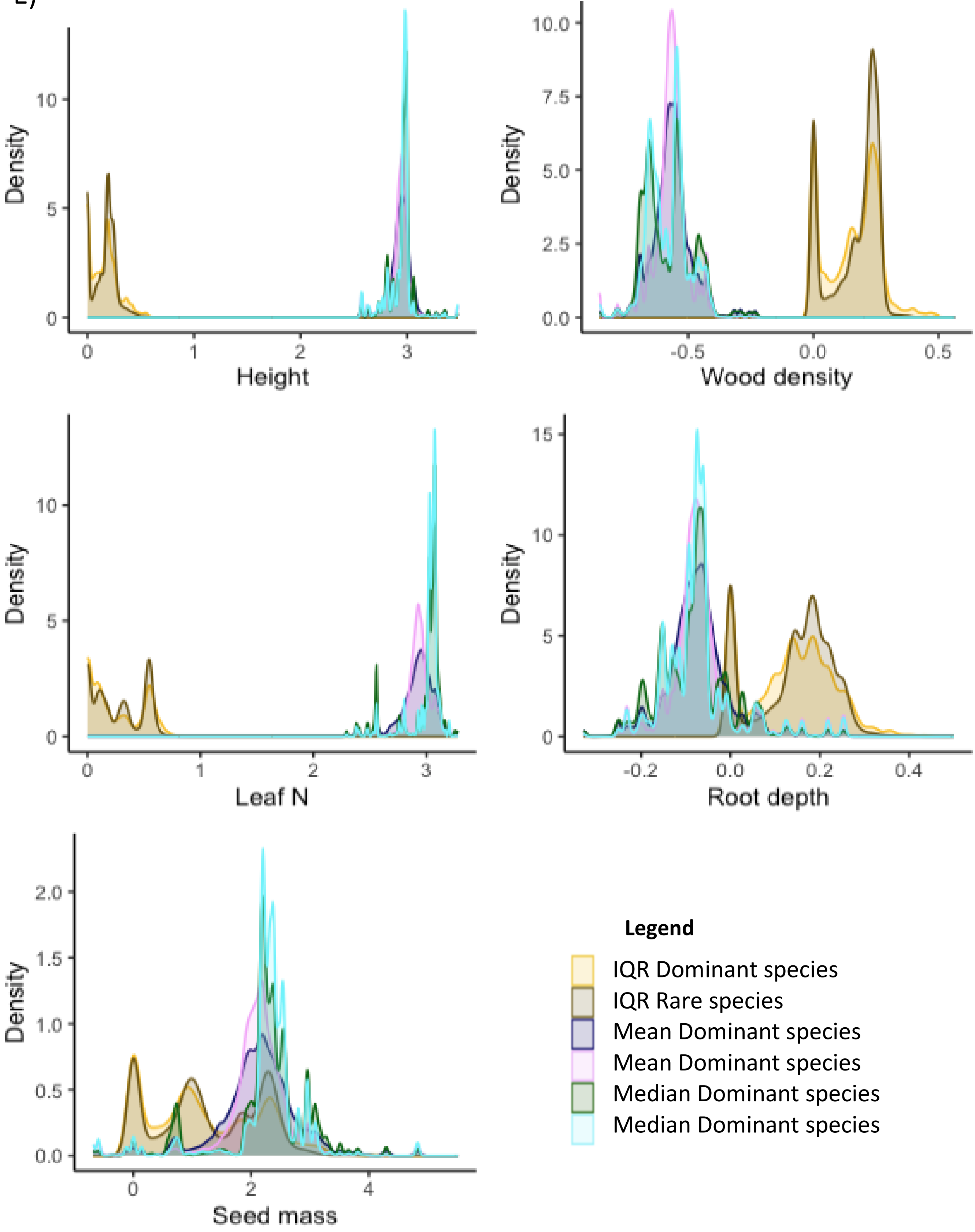

Figure S4. The visualisation of the density of the log10-transformed trait values for dominant and rare tree trait values per plot, calculated as the mean, median and interquartile range. The dominant and rare species were based on a 100 times randomization of the five main forest biomes individually (A-E). In this randomization process, the total number of trees per plot and the total number of individuals per tree species across the biome was held constant. Afterwards, we calculated the traits of the dominant and rare species in three different ways: using the trait mean, the median and the interquartile range. The trait values were based on the mean trait value of the individual species per biome. From the visualisations of the randomizations, we can conclude that calculating the plot level trait values of the dominant and rare species using interquartile ranges differs most compared to calculating the plot level trait values using the mean or median value. The interquartile range usually shows a peak in frequency at (close to) zero, indicating that there is only one dominant or rare species identified or that trait values between dominant or rare species are very similar. The median shows more variation in trait values compared to the mean, especially in less species rich biomes, but trait values largely overlap. There is a large to complete overlap of frequency of trait values between dominant and rare species for the three different ways of calculating traits of dominant and rare species at plot level. The more species a biome harbours, and therefore the more random the randomization becomes, the more complete the overlap is.

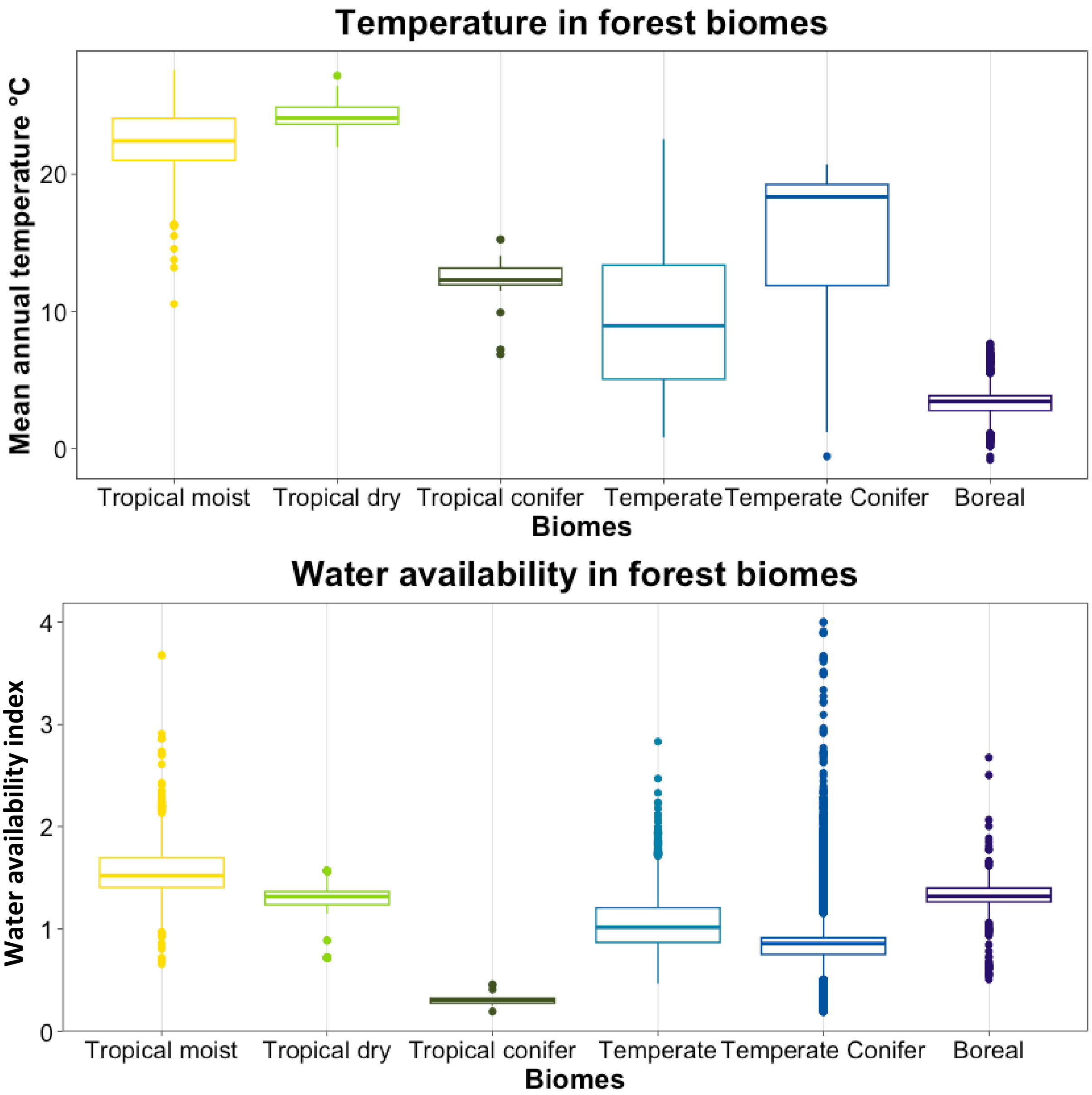

Figure S5. An overview of the mean annual temperature (upper panel) and water availability (lower panel) in the six different forest biomes: tropical moist forest (1694), tropical dry forest (1015), tropical conifer forest (20), temperate forest (9721), temperate conifer forest (8813) and boreal forest (1562). The median, 25th and 75th percentiles of the data are visualised with the boxplot, while the whiskers present the main data range.

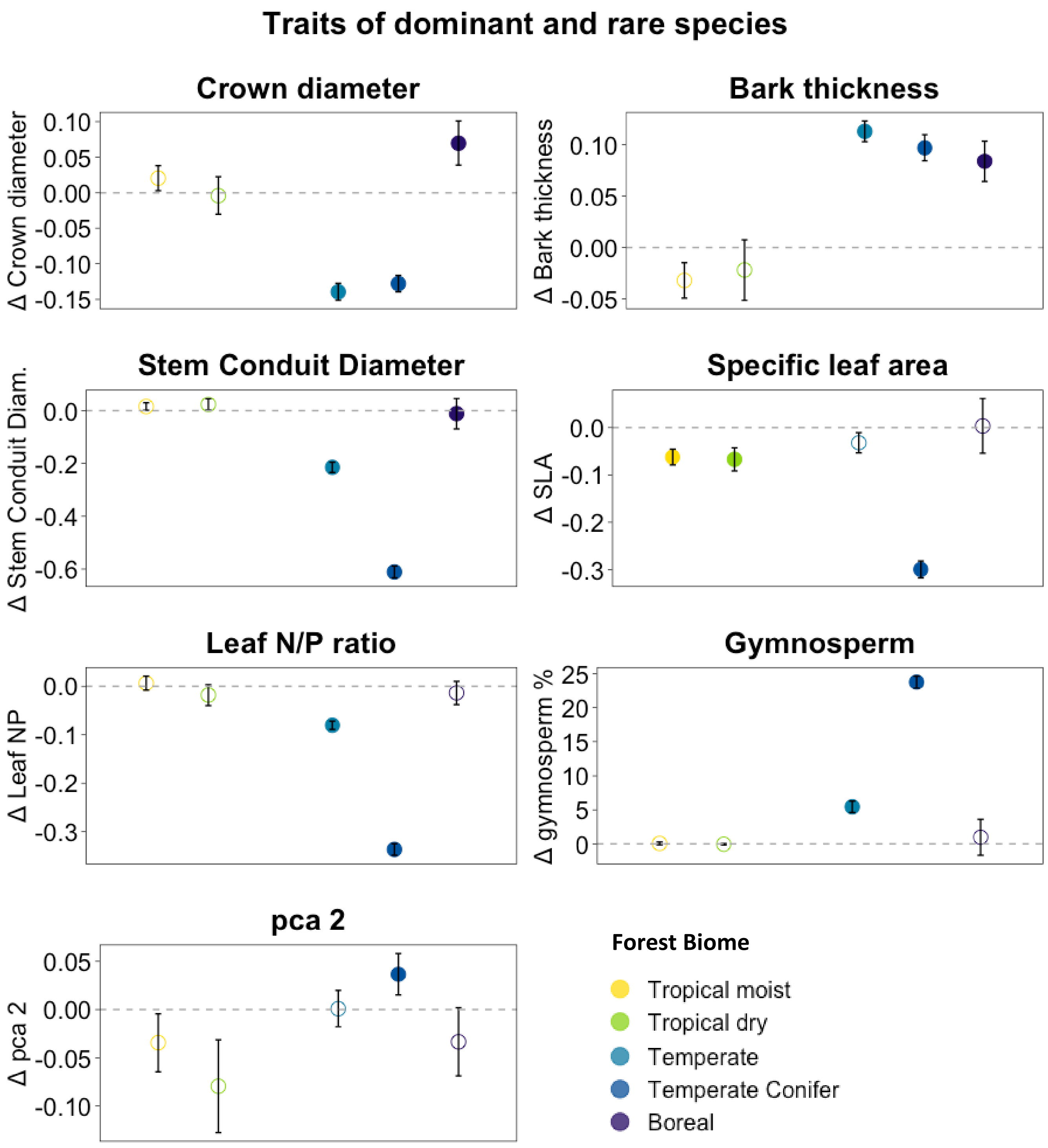

Figure S6. The difference between standardized trait values of dominant and rare species per forest biome. Ten traits and the first two PC axes are evaluated, of which five traits, the percentage classified as gymnosperms and PC2 are visualized here. The mean and standard error of the difference between trait values of dominant and rare species are displayed. If the mean is positive, dominant species have on average a higher trait value, while a negative mean indicates that rare

species have a higher trait value. The grey dashed line indicates similar trait values between dominant and rare species. Closed dots indicate a significant different between trait values of dominant and rare species (Wilcoxon test, $N>1015$, $p<0.05$).

Trait differences correlated with climate

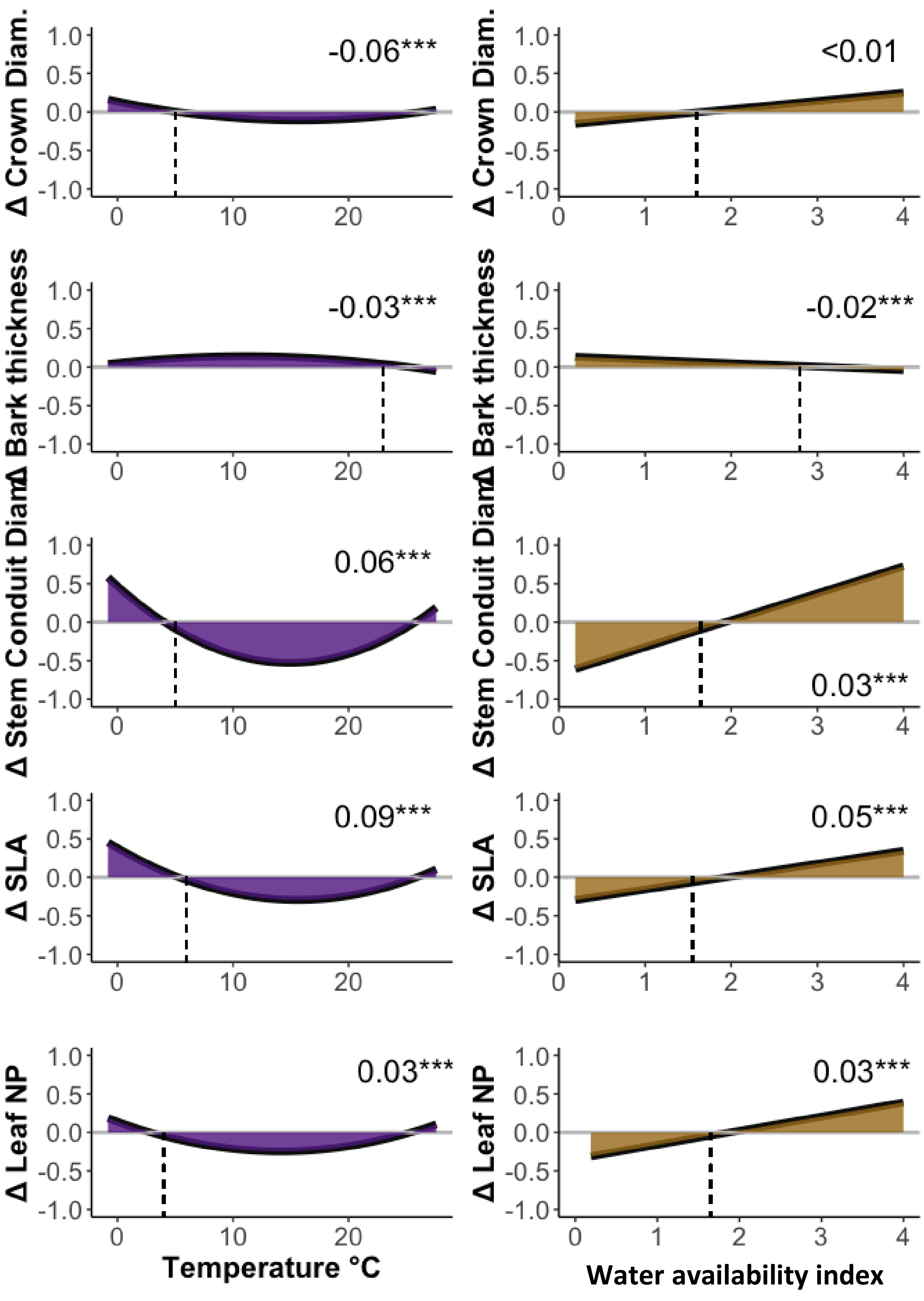

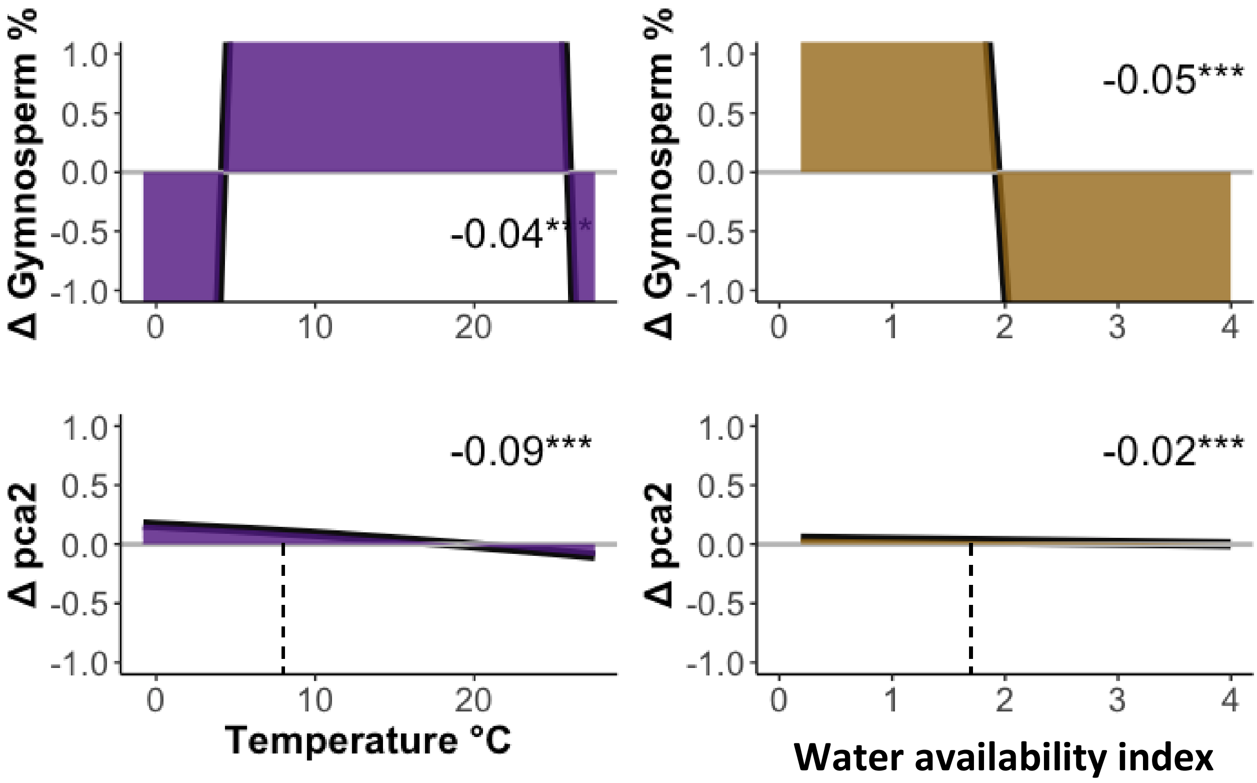

Figure S7. The difference between six dominant and rare trait values and PC2 at the y-axis plotted against respectively mean annual temperature (left) and water availability (right). Positive values indicate that trait values are higher for dominant species, while negative values indicate that trait values are higher for rare species. The graphs are modelled with a second order polynomial function, with the 95% confidence interval indicated in grey. Number of plots included in this analysis is 22825. The standardized regression coefficient is displayed, and its significance is indicated with asterisks: *p<0.05, **p<0.01, ***p<0.001.

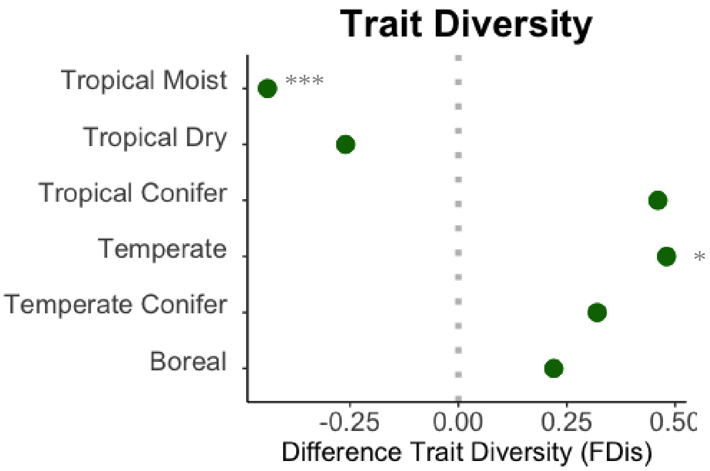

Figure S8. Difference in trait diversity of the dominant and rare species per biome. At the biome level, all tree individuals were summed up and the 10% most dominant and rare species were identified based on the number of stems. Trait diversity is defined as trait dispersion (FDis) (Laliberté & Legendre, 2010). FDis is the weighted mean distance in multidimensional trait space of individual species to the weighted centroid of all species, where weights correspond to the relative abundances of the species (Laliberté & Legendre, 2010). Positive value indicates that dominant species have a higher trait diversity value, while a negative value indicates that rare species have a higher trait diversity value. The asterisks express a significant difference between respectively the centroids or trait diversity of the dominant and rare species per forest biome (* $p<0.05$, **$p<0.01$, ***$p<0.001$).

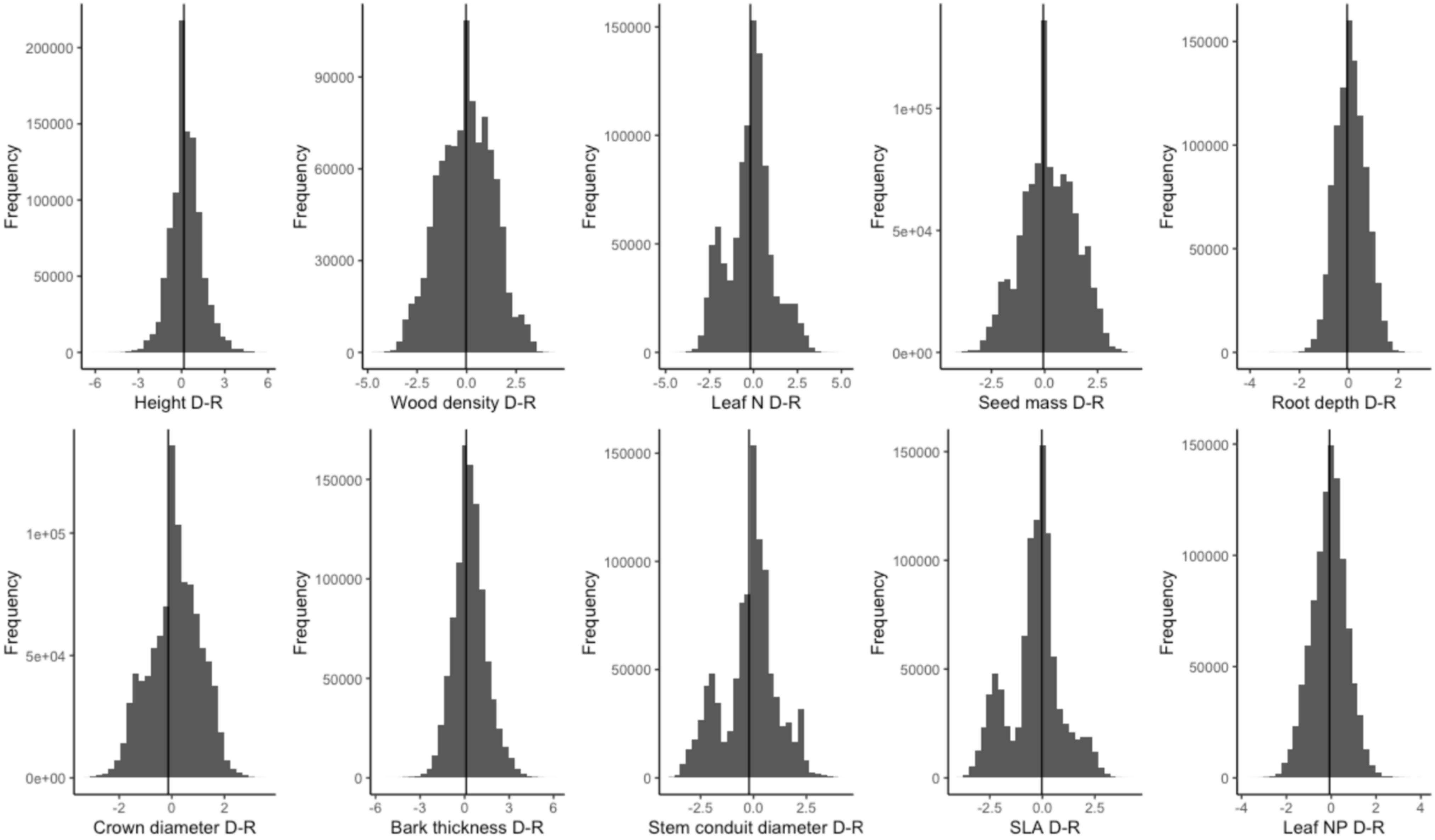

Figure S9. Bootstrapping results for selection of plots in the temperate forest biome. At the y-axis the frequency of observation is visualised, while at the x-axis the difference between the dominant and rare trait value is depicted. The mean difference between the dominant and rare trait values, as used in the

main analyses, are visualised with a vertical line. In the temperate forest biome, we have a total of 135,043 plots included in the database. To have a more balanced dataset, we filtered 10.000 plots. We performed a bootstrapping analysis, in which we 100 times randomly select 10.000 plots of the temperate forest biome for all single traits. The bootstrapping procedure shows that the differences between dominant and rare traits we use in our main text aligns with the peak of the difference between the trait values according to the bootstrapping results.

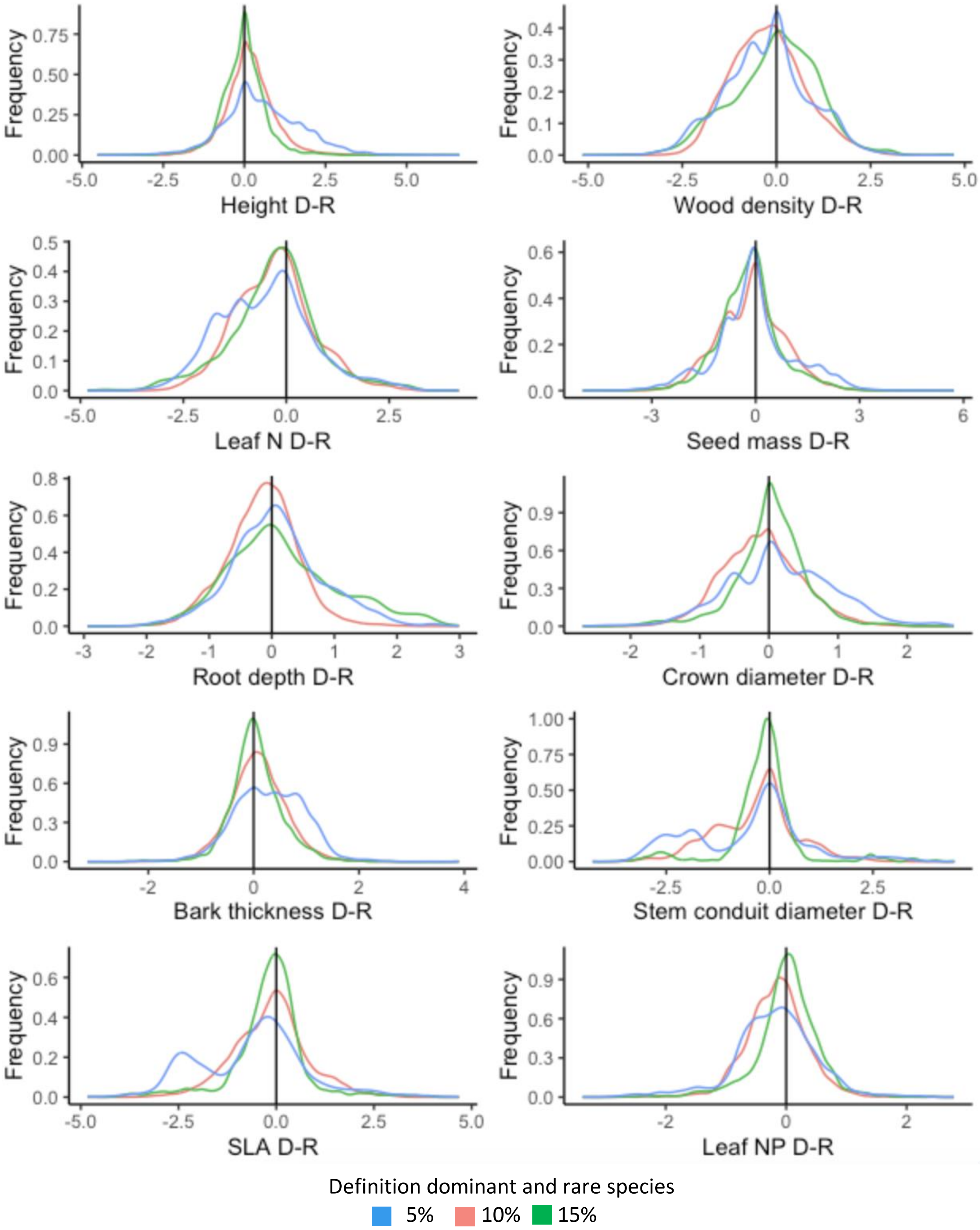

Figure S10. Results of different definitions of dominant and rare species for difference in trait values.

At the y-axis the frequency of observations and the x-axis the difference between dominant (D) and

rare (R) trait values for the difference traits. In this analysis we defined the dominant and rare species, as respectively the species with the top and bottom 5% (blue), 10% (pink) and 15% (green) of individuals in the plot. When defining dominant and rare species using the 10% definition, as in the main text, 26% of the database was included. When defining dominant and rare species using the 15% definition, 27% of the database was included. For the different definitions of dominant and rare species, the difference for the individual traits was calculated.

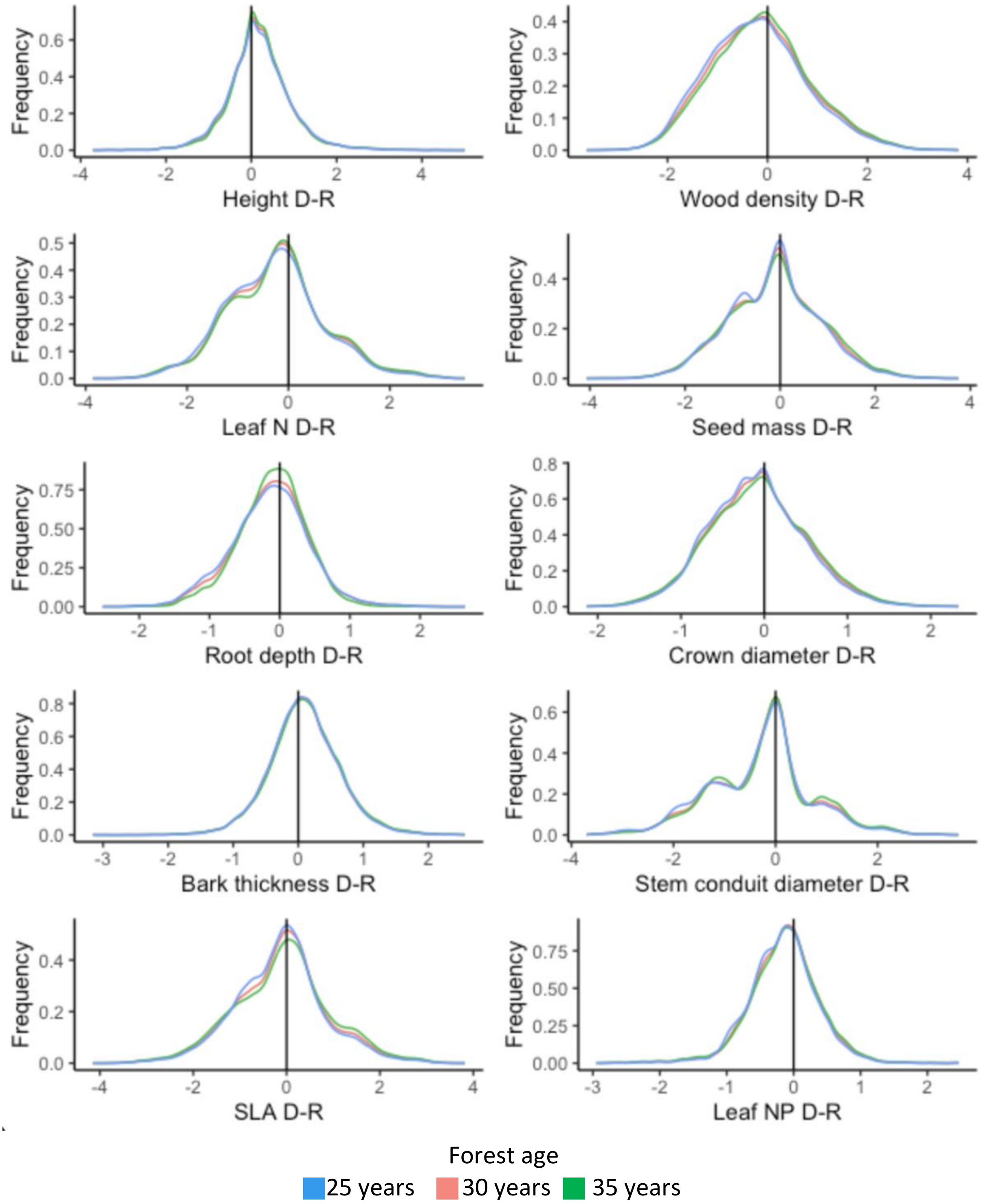

Figure S11. Results of different filters of forest age for difference in trait values. At the y-axis the frequency of observations and the x-axis the difference between dominant (D) and rare (R) trait values for the difference traits. In this analysis we applied different forest age filters, 25 years (blue), 30 years (pink) and 35 years (green). When defining dominant and rare species using the 30 years

threshold, 83% of the database was included. When defining dominant and rare species using the 35 years threshold, 64% of the database was included. For the different forest age filters, the difference in trait values for the dominant and rare species for the individual traits was calculated.

Tables

Table S1. The p-values and test statistics of the Wilcoxon tests to evaluate differences between trait values of dominant and rare tree species per biome.

**Biome**	Tropical moist forest	Tropical dry forest	Tropical conifer forest	Temperate forest	Temperate conifer forest	Boreal forest
**N**	1694	1015	20	9721	8813	1516
**Pvalue_Height**	0.0015	0.0027	0.0907	<0.0001	<0.0001	<0.0001
**Pvalue_CrownDiameter**	0.6216	0.8433	0.2555	<0.0001	<0.0001	0.0031
**Pvalue_BarkThickness**	0.4363	0.7514	0.0256	<0.0001	<0.0001	<0.0001
**Pvalue_WoodDensity**	<0.0001	<0.0001	0.0047	<0.0001	<0.0001	<0.0001
**Pvalue_SLA**	<0.0001	<0.0001	0.0001	0.1811	<0.0001	0.5591
**Pvalue_LeafN**	0.0876	0.0088	0.0001	<0.0001	<0.0001	0.0333
**Pvalue_LeafNP**	<0.0001	0.2085	0.0023	<0.0001	<0.0001	0.6228
**Pvalue_StemConduitDiameter**	0.1442	0.0728	<0.0001	<0.0001	<0.0001	0.0257
**Pvalue_RootDepth**	0.1086	0.0514	0.0040	<0.0001	<0.0001	0.0006
**Pvalue_SeedMass**	0.1098	0.0001	0.0009	0.0035	<0.0001	<0.0001
**Pvalue_Gymnosperm**	0.9976	0.3178	0.0010	<0.0001	<0.0001	0.9473
**Pvalue_pca1**	0.0099	0.0001	0.0003	<0.0001	<0.0001	0.0001
**Pvalue_pca2**	0.2066	0.0664	0.3792	0.4782	<0.0001	0.2306
**Wstatistics_Height**	1344376.5	475548.5	263	39650337	34278987.5	980838.5
**Wstatistics_CrownDiameter**	1420765.5	517724	242.5	53323751	47229821.5	1145281.5
**Wstatistics_BarkThickness**	1456982	519296	283	40405349	35983486	1086790
**Wstatistics_WoodDensity**	1564875.5	570861	95	49441879	53798799.5	1477354

**Wstatistics_SLA**	1579689.5	587464.5	52	47772241	47709646.5	1205198
**Wstatistics_LeafN**	1483443	549728	54	54544336	55498637.5	1166274
**Wstatistics_LeafNP**	1419016	531719	87	51061436	52087582.5	1232323
**Wstatistics_StemConduitDiameter**	1393247.5	491425	45	56055893	53715310.5	1163685
**Wstatistics_RootDepth**	1480497.5	489388	93	49369516	49006666	1306032
**Wstatistics_SeedMass**	1389291.5	464101	77	48393039	48049596.5	1457426
**Wstatistics_Gymnosperm**	1434809.5	515620	320	43205128.5	26120534.5	1221553
**Wstatistics_pca1**	1361414.5	463233.5	333	39237289	23896825.5	1122493
**Wstatistics_pca2**	1470776.5	539351	167	47526399	36992265.5	1250140
