# Supplementary material for: Effect of climate on traits of dominant and rare tree species in the world’s forests
Source: Nat Commun. 2025 May 22;16:4773. doi: 10.1038/s41467-025-59754-7 (PMC12098762; doi:10.1038/s41467-025-59754-7)
Supplement: Supplementary file 3 — Description of Additional Supplementary Files [file 41467_2025_59754_MOESM3_ESM.pdf]

Description of Additional Supplementary Files

File Name: Supplementary Data 1
Description: Regression coefficients and variable importance of the independent variables, evaluated with linear models. To quantify the relative importance of temperature, water availability index (WAI), their interaction, forest age, plot size, elevation and biome on trait differences, we used the scaled calc.relimp function in R (Grömping, 2006). This function evaluates the contribution of each independent variable to the variation explained, by averaging the contribution of each independent variable to the r2 in terms of its sum of squares across all possible fitting sequences. The number of plots incorporated in the analysis is 22825.

File Name: Supplementary Code 1
Description: This code is used to produce the results and figures in the manuscript titled: "Effect of climate on traits of dominant and rare tree species in the world's forests". The data is available to reproduce the main graphs. This R markdown file is annotated, to guide the reader through the code and different steps in the analyses. A detailed description of the methods can be found in the methods section of the manuscript.
