# Supplementary material for: Effect of climate on traits of dominant and rare tree species in the world’s forests
Source: Nat Commun. 2025 May 22;16:4773. doi: 10.1038/s41467-025-59754-7 (PMC12098762; doi:10.1038/s41467-025-59754-7)
Supplement: Supplementary file 6 — Reporting Summary [file 41467_2025_59754_MOESM6_ESM.pdf]

nature portfolio

Corresponding author(s): DAPR

Last updated by author(s): Dec 21, 2024

Reporting Summary

Nature Portfolio wishes to improve the reproducibility of the work that we publish. This form provides structure for consistency and transparency in reporting. For further information on Nature Portfolio policies, see our Editorial Policies and the Editorial Policy Checklist.

Statistics

For all statistical analyses, confirm that the following items are present in the figure legend, table legend, main text, or Methods section.

n/a	Confirmed	
☐	☒	The exact sample size (*n*) for each experimental group/condition, given as a discrete number and unit of measurement
☐	☒	A statement on whether measurements were taken from distinct samples or whether the same sample was measured repeatedly
☐	☒	The statistical test(s) used AND whether they are one- or two-sided<br>*Only common tests should be described solely by name; describe more complex techniques in the Methods section.*
☐	☒	A description of all covariates tested
☐	☒	A description of any assumptions or corrections, such as tests of normality and adjustment for multiple comparisons
☐	☒	A full description of the statistical parameters including central tendency (e.g. means) or other basic estimates (e.g. regression coefficient) AND variation (e.g. standard deviation) or associated estimates of uncertainty (e.g. confidence intervals)
☐	☒	For null hypothesis testing, the test statistic (e.g. *F*, *t*, *r*) with confidence intervals, effect sizes, degrees of freedom and *P* value noted<br>*Give P values as exact values whenever suitable.*
☒	☐	For Bayesian analysis, information on the choice of priors and Markov chain Monte Carlo settings
☒	☐	For hierarchical and complex designs, identification of the appropriate level for tests and full reporting of outcomes
☐	☒	Estimates of effect sizes (e.g. Cohen's *d*, Pearson's *r*), indicating how they were calculated

Our web collection on statistics for biologists contains articles on many of the points above.

Software and code

Policy information about availability of computer code

Data collection	No software was used
Data analysis	R-Studio interface to R. R Studio version Version 2024.09.1+394. R version 4.4.0.

For manuscripts utilizing custom algorithms or software that are central to the research but not yet described in published literature, software must be made available to editors and reviewers. We strongly encourage code deposition in a community repository (e.g. GitHub). See the Nature Portfolio guidelines for submitting code & software for further information.

Data

Policy information about availability of data

All manuscripts must include a data availability statement. This statement should provide the following information, where applicable:

- Accession codes, unique identifiers, or web links for publicly available datasets
- A description of any restrictions on data availability
- For clinical datasets or third party data, please ensure that the statement adheres to our policy

The plot-level data of the difference between trait values of dominant and rare tree species will be stored upon acceptance in the Research Data Repository of the Research Collection of ETH Zürich. The GFBI database is available upon written request at https://gfbinitiative.net/data/. Data management and statistical analyses in this study were performed with the R-Studio interface to R (R Core Team, 2024). The code used to perform the statistical analyses can be found at Github, following this link: https://tinyurl.com/2jxb8555

Research involving human participants, their data, or biological material

Policy information about studies with human participants or human data. See also policy information about sex, gender (identity/presentation), and sexual orientation and race, ethnicity and racism.

Reporting on sex and gender	NA
Reporting on race, ethnicity, or other socially relevant groupings	NA
Population characteristics	NA
Recruitment	NA
Ethics oversight	NA

Note that full information on the approval of the study protocol must also be provided in the manuscript.

Field-specific reporting

Please select the one below that is the best fit for your research. If you are not sure, read the appropriate sections before making your selection.

☐ Life sciences ☐ Behavioural & social sciences ☒ Ecological, evolutionary & environmental sciences

For a reference copy of the document with all sections, see nature.com/documents/nr-reporting-summary-flat.pdf

Ecological, evolutionary & environmental sciences study design

All studies must disclose on these points even when the disclosure is negative.

Study description	Forest inventory of 1.2 million forest plots around the globe.
Research sample	NA
Sampling strategy	NA
Data collection	Data on DBH, height and species of trees were collected by the co-authors of this manuscript (GFBI community).
Timing and spatial scale	The data is collected between 1950 and 2017
Data exclusions	Plot sizes range from 0.0002 to 20 ha in the database and the plots include all trees with stem diameter at breast height > 5 cm. As rare species are likely not captured accurately in very small plots, and trait variation is correlated with plot size as well (Karadimou et al., 2016), we excluded plot sizes smaller than the first quantile of 0.02 ha and outliers larger than 2 ha (in total 8.3% of the database). Additionally, plots measured before 1990 were filtered out, as these plots likely do not represent current forest composition and do not match with the climatic data we used (filtering out 21% of the remaining database). Also, trait values of the rare and dominant species change with successional forest age (Lohbeck et al., 2014), we therefore excluded early successional plots with a forest age of less than 25 years, which corresponds to 1.3% of the remaining database (Poulter et al., 2019). Different forest age thresholds could potentially affect trait values of dominant and rare species. Yet, when comparing trait values using the 25-year threshold with 30- and 35-years thresholds we got very similar results (Fig. S11). Within the filtered database, the correlations between plot size and number of dominant species ($r=0.23$, $r2=0.05$, $p<0.01$), and plot size and number of rare species was equally weakly related ($r=0.23$, $r2=0.05$, $p<0.01$) (see for definition of dominant and rare species in section 2.2). See for an overview of the distribution of plot size within every forest biome Fig. S1 and the relationship between species abundances and plot size for this database Hordijk et al. (2023) and Hordijk et al. (2024). In our filtered dataset, mean plot size is 0.07 ha, mean measurement year is 2004 and 53 years is the mean forest age, whereas 1.4% of the dataset is made up of old growth forest (older than 140 years). Elimination of forest plots based on size, year of measurement, forest age, and incorporating only plots within the forest biomes (Dinerstein et al., 2017), resulted in 660,552 plots in the filtered dataset (Fig. 1). Additionally, we incorporated only plots with six or more species, to clearly separate dominant and rare species, which included 23% of the filtered dataset based on plot size, measurement year and age.
Reproducibility	NA
Randomization	NA
Blinding	NA

Did the study involve field work? ☒ Yes ☐ No

Field work, collection and transport

Field conditions	The GFBI database consists of forest inventory information of 1.2 million forest plots around the globe.
Location	The GFBI database consists of forest inventory information of 1.2 million forest plots around the globe.
Access & import/export	No samples were exported or imported.
Disturbance	Minimal ecological disturbance is expected by measuring the trees in a forest.

Reporting for specific materials, systems and methods

We require information from authors about some types of materials, experimental systems and methods used in many studies. Here, indicate whether each material, system or method listed is relevant to your study. If you are not sure if a list item applies to your research, read the appropriate section before selecting a response.

Materials & experimental systems

n/a	Involved in the study
☒	☐ Antibodies
☒	☐ Eukaryotic cell lines
☒	☐ Palaeontology and archaeology
☒	☐ Animals and other organisms
☒	☐ Clinical data
☒	☐ Dual use research of concern
☒	☐ Plants

Methods

n/a	Involved in the study
☒	☐ ChIP-seq
☒	☐ Flow cytometry
☒	☐ MRI-based neuroimaging

Plants

Seed stocks	NA
Novel plant genotypes	NA
Authentication	NA
